# Disruptive selection and the evolution of discrete color morphs in *Timema* stick insects

**DOI:** 10.1126/sciadv.abm8157

**Published:** 2023-03-31

**Authors:** Romain Villoutreix, Clarissa F. de Carvalho, Jeffrey L. Feder, Zachariah Gompert, Patrik Nosil

**Affiliations:** ^1^ CEFE, Université Montpellier, CNRS, EPHE, IRD, Montpellier, France.; ^2^ Departamento de Ecologia e Biologia Evolutiva, UNIFESP, Diadema 09972-270, Brazil.; ^3^ Department of Biological Sciences, University of Notre Dame, Notre Dame, IN 46556, USA.; ^4^ Department of Biology, Utah State University, Logan, UT 84322, USA.

## Abstract

A major unresolved issue in biology is why phenotypic and genetic variation is sometimes continuous, yet other times packaged into discrete units of diversity, such as morphs, ecotypes, and species. In theory, ecological discontinuities can impose strong disruptive selection that promotes the evolution of discrete forms, but direct tests of this hypothesis are lacking. Here, we show that *Timema* stick insects exhibit genetically determined color morphs that range from weakly to strongly discontinuous. Color data from nature and a manipulative field experiment demonstrate that greater morph differentiation is associated with shifts from host plants exhibiting more continuous color variation to those exhibiting greater coloration distance between green leaves and brown stems, the latter of which generates strong disruptive selection. Our results show how ecological factors can promote discrete variation, and we further present results on how this can have variable effects on the genetic differentiation that promotes speciation.

## Teaser

Higher color difference between the leaves and stems of plants selects for discrete body coloration in stick insects.

## INTRODUCTION

Phenotypic and genetic variation in nature is regularly packaged into discontinuous forms such as discrete morphs or species (i.e., divergent forms with few intermediates) (*1*–*5*). However, many other times, variation is more continuous and unimodal, as occurs for quantitative traits (e.g., body size) in many species (*3*). Understanding the causes of these variable outcomes is fundamental for understanding the conditions associated with the evolution of discrete units of biodiversity (*5*, *6*).

Much work has already been done to understand the emergence of new forms, particularly in the context of the formation of new species (*6*–*8*). In this context, the effects of geographical barriers on reducing gene flow between populations and promoting the creation of distinct species have long been recognized (*6*, *9*). More recently, many studies focused on cases where speciation happens in the absence of geographic barriers, with ongoing gene flow (*6*, *8*). Under this scenario, disruptive selection for different niches, habitats, or strategies can promote phenotypic divergence and reproductive isolation (*8*). Thus, there is much current interest in the role of disruptive selection in the emergence of discrete units of diversity (e.g., new phenotypic forms and ultimately new species). A role for disruptive selection in the emergence of different phenotypic forms and new species has been inferred in many systems (*10*, *11*), but we still lack a clear understanding of the strength of selection needed to create such divergent discrete forms and details of the ecological factors causing the strength of divergent selection to vary.

In this context, a number of genetic and ecological factors can contribute to trait discontinuity. In terms of genetic factors, both the number of loci controlling a trait and the degree of genetic control will affect the phenotypic distribution and thus the discontinuity of a trait. Traits controlled by many recombining loci and/or with a low degree of genetic control should tend to have a more continuous distribution than those controlled by a major locus of large effect, particularly if the latter exhibit genetic dominance (*1*). In addition, the degree of continuity of traits can be influenced by ecological factors. For example, traits may vary quantitatively because a wide range of variation allows for high fitness in certain ecological conditions (*2*, *12*, *13*). Alternatively, ecological factors may generate selection against intermediates (i.e., disruptive selection), promoting discrete variation. Specifically, sharp, discontinuous transitions in ecological variables (ecological discontinuities, hereafter) may generate strong disruptive selection that leads the evolution of discrete forms (*14*) (we refer to this hypothesis as the “ecological discontinuity hypothesis” hereafter).

The ecological discontinuity hypothesis generates two core predictions. First, the discreteness of genetically controlled traits should covary with the degree of ecological discontinuity in the environment (i.e., stronger ecological discontinuities will be associated with more discontinuous traits). Second, the strength of disruptive selection will vary between environments (i.e., stronger ecological discontinuities generate stronger disruptive selection). Although the ecological discontinuity hypothesis is intuitive and had been discussed previously (*2*, *13*), direct tests of its predictions are lacking. This is because ecological variation and the strength of selection are often inferred and not directly and experimentally measured, or because studies focus on one particular level of discontinuity rather than a range of it (*14*–*18*). Here, we directly test these predictions using natural history observations of both insects and plants. We also perform a manipulative field experiment to verify that the ecological discontinuity that we identified affects disruptive selection and thus trait discontinuity. Last, we use genomic data to test the consequences of variable phenotypic differentiation for the genetic differentiation that can generate reproductive isolation, a key component of species formation (*5*, *6*). Although the focus of this study is on color morphs, processes similar to those described and studied here could explain the emergence of other recognizable units of diversity, such as ecotypes or species (*5*, *6*).

### Study system: *Timema* stick insects and their host plants

**Fig. 1.**
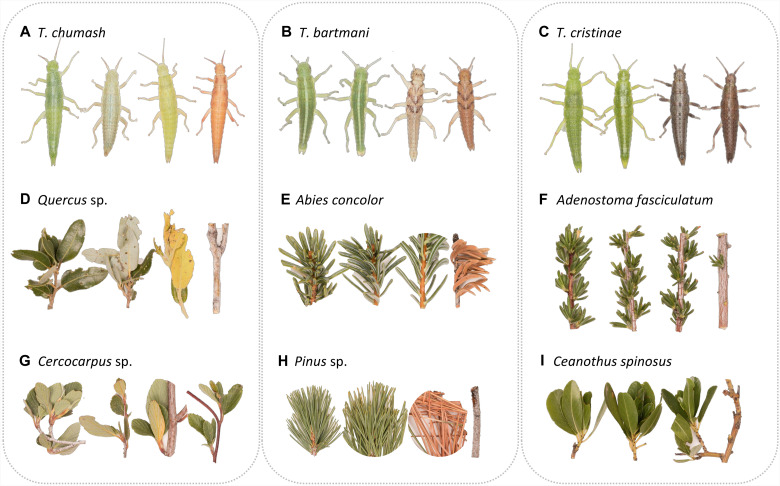
Photographs depicting representative coloration of different *Timema* species and their main host plants. For *T. chumash* (**A**), the main host plants are oak [*Quercus* sp.; (**D**)] and mountain mahogany [*Cercocarpus* sp.; (**G**)]; for *T. bartmani* (**B**), the main host plants are white fir [*A. concolor*; (**E**)] and white pine [*Pinus* sp.; (**H**)]: for *T. cristinae* (**C**), the main host plants are chamise [*A. fasciculatum*; (**F**)] and California lilac [*C. spinosus*; (**I**)].

In this work, we study wingless, herbivorous *Timema* stick insects, which rely on crypsis for protection against visual predators while resting on their host plants (*19*–*23*). Most species exhibit body color polymorphisms, with green and darkly colored individuals, the latter having tones of brown, red, or gray (referred to as melanistic individuals hereafter, following past work) (Fig. 1) (*19*, *21*, *24*–*26*). Natural selection from visually oriented predators, such as birds and lizards, is likely the main mechanism maintaining this polymorphism (*21*) and may have driven its emergence. Specifically, green individuals are more cryptic on leaves, while melanistic individuals are more cryptic on darkly colored stems (*19*, *21*). Green and melanistic individuals are found on the same individual plant in most species and readily mate with one another in laboratory trials (*19*, *25*, *26*). There is some evidence for a mating advantage for melanistic individuals in the one species studied to date (*Timema cristinae*), but no evidence for assortative mating per se (*19*). Moreover, the aforementioned mating advantage is unlikely to be caused directly by color, as *Timema* use chemical cues (i.e., cuticular hydrocarbons) for mate choice (*27*, *28*). Last, past studies have shown evidence of frequency-dependent selection for body pattern (i.e., the presence or absence of a white stripe on the dorsal side of green individuals) but not for body coloration in *Timema* (*20*). Frequency-dependent selection for body pattern is likely driven by bird predation and associated predator search images (*20*).

In this study, we focus on three *Timema* species that differ in the degree of discontinuity for their green and melanistic morphs (*26*). Specifically, body coloration is highly discontinuous in two species (*Timema bartmani* and *T. cristinae*), but more continuous in a third (*Timema chumash*). Nonetheless, as we show below, coloration in all these species is bimodal, allowing for the identification of green and melanistic morphs in all three species (and we also present below information on intermediate morphs). Previous studies showed that body coloration is highly heritable (*19*, *20*, *24*, *26*) but associated with different genetic architectures (*26*). Specifically, body coloration is associated with multiple linked yet recombining loci in a collinear genomic region in *T. chumash*. In contrast, coloration is associated with a region of reduced recombination (i.e., the *Mel-Stripe* locus; a putative supergene where recombination is strongly suppressed) in *T. barmani* and *T. cristinae* (*25*, *26*, *29*). While previous studies focused on the genetic factors associated with variation in body coloration discontinuity, this study focuses on identifying the ecological factors (here plant coloration) that act on body coloration discontinuity.

For *Timema*, the ecological discontinuity hypothesis predicts that greater discontinuity in the colors between leaves versus stems of host plants will be associated with greater discontinuity in color between green versus melanistic morphs (*30*–*32*). We tested this prediction by studying the three *Timema* species mentioned above (Fig. 1). While we observed informally in past work that these species exhibit different degrees of discontinuity for their green and melanistic morphs, morph discontinuity has not previously been quantified and compared among species in a formal manner. The present study quantifies such discontinuity and compares it with the difference in color between leaves versus stems of their host plants. We also conducted a new release-recapture experiment to test the selective regime and strength of selection imposed by different hosts and, especially, to measure the fitness of individuals with intermediate coloration. While past work in *T. chumash* showed that the selection regime on body coloration is host plant dependent (*25*), this work was not designed to specifically test the fitness of individuals with intermediate coloration, because these were at low (i.e., naturally occurring) frequencies in the experiment. We thus designed a new experiment, better suited to testing the fitness of individuals with intermediate coloration on different host plants (by enriching the trait distribution for intermediate coloration; details below), and thus the ecological discontinuity hypothesis.

## RESULTS

### *Timema* and their host plants reflect mainly in the visible spectrum

Our study focuses primarily on the visible spectrum (i.e., the portion of the electromagnetic spectrum that is visible to the human eye). To test whether this focus was justified, we analyzed spectral reflectance data for *Timema* and their host plants using methods similar to those that we used in past work (*26*). We found that all *Timema* specimens reflect mainly in the visible spectrum and exhibit low reflectance at ultraviolet wavelengths (between 300 and 400 nm; with average reflectance below 6%; fig. S1). Similar patterns were found for the leaves and stems of host plants, with the exception of a marginal ultraviolet reflectance for the leaves of *Abies concolor* (fig. S2).

Although it is common to disregard ultraviolet spectra with less than 10% reflectance (*33*–*36*), we nonetheless evaluated how the reflectance of *Timema* and their host plants was perceived by birds. Specifically, we modeled the reflectance of *Timema* and their host plants in the context of the avian visual system (*37*, *38*) and found that reflectance in the ultraviolet range only minimally contributes to the perception of *Timema* and host plant coloration by birds (the modeled perception of colors in the visible spectrum is, on average, 97% for *Timema* and 96% for their host plants; see Materials and Methods for details; figs. S3 and S4). Collectively, these results showed that *Timema* and their host plants mostly reflect colors in the visible spectrum and that this range is what is mostly seen by birds, their main predators. Thus, our focus on the visible color spectrum is relevant biologically. Moreover, the main objective of our work is to evaluate whether color difference in *Timema* is associated with color difference in their host plants and not to quantify *Timema* background matching per se. In addition, recent studies have shown that human vision can be a valid proxy for avian perception of color differences, giving qualitatively similar results to avian models (*39*, *40*).

### *Timema* have green and melanistic morphs whose differentiation varies between species

**Fig. 2.**
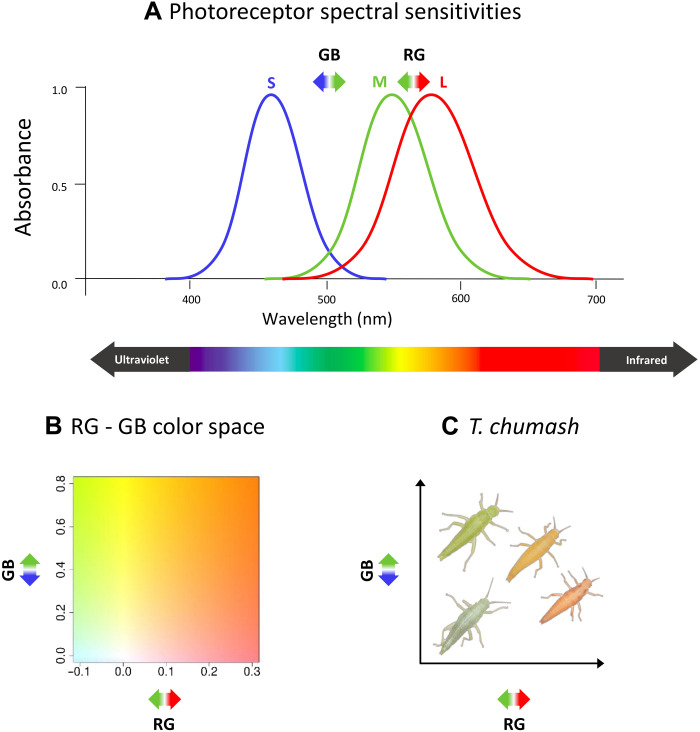
A schematic representation of coloration measurements used in this study. (**A**) Schematic spectral sensitivities of human photoreceptors. The human visible spectrum represents cones that capture long wavelengths (L, red), medium wavelengths (M, green), and short wavelengths (S, blue). Differences between L-M and M-S activities are the most common responses in color perception (*76*). Relative differences between values of red and green (RG) and of green and blue (GB) extracted from digital photographs can be used as an approximation to this physiological response, which represents the great majority of *Timema*’s color variation (see results on spectral reflectance). (**B**) RG and GB are orthogonal measures and together capture the range of color variation observed in *Timema* coloration. The graphic depicts the color associated with given RG and GB values. We focus here on the range of RG and GB values observed in our study. (**C**) *T. chumash* individuals displayed in the RG and GB color space. Figure was modified from (*25*). For explicit consideration of the avian visual system and ultraviolet light, see Results (“*Timema* and their host plants reflect mainly in the visible spectrum” section) and Materials and Methods.

Following past work (*24*–*26*), we used standardized photos of 881 individuals to quantify body coloration in *Timema*. Specifically, we recorded mean red, green, and blue (RGB) color values, with which we calculated chromatic contrasts as the relative difference between: (i) red and green color (which contrasts long and medium wavelengths in the visible spectrum; a trait referred to as “RG” hereafter; Fig. 2; see Materials and Methods for details on measurements and methods) and (ii) between green and blue color (which contrasts medium and short wavelengths in the visible spectrum; a trait referred to as “GB” hereafter; Fig. 2) (*41*). These RG and GB estimates allowed us to quantitatively describe color in a two-dimensional plane (the RG-GB color space, hereafter; Fig. 2B).

**Fig. 3.**
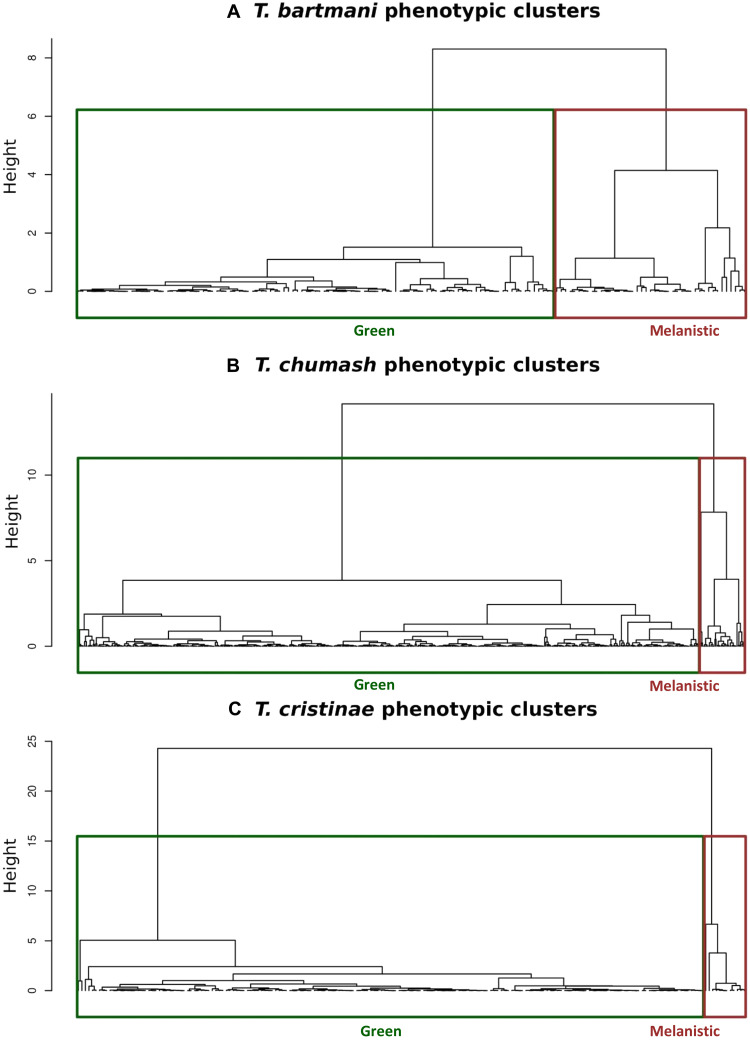
Evidence of color morphs from hierarchical clustering. Each panel shows the UPGMA tree from pairwise color distances among individuals within species: (**A**) *T. bartmani*, (**B**) *T. chumash*, and (**C**) *T. cristinae*. The identified hierarchical clusters are denoted with green (green morph) and brown (melanistic morph) boxes. Comparable results were observed using other methods, as described in Results (“*Timema* have green and melanistic morphs whose differentiation varies between species” section).

Using the RG and GB estimates, we confirmed by hierarchical clustering [through the unweighted pair group method with arithmetic mean method (UPGMA)] that all the species in this study have identifiable green and melanistic morphs, which we defined using the results of the UPGMA method (Fig. 3; see Materials and Methods for details). We found additional support for the existence of distinct green and melanistic morphs in the three species via mixture modeling. These analyses compared the fit of models to a single (i.e., unimodal) distribution versus those involving a mixture of two distributions. The results showed that color in *Timema* was best described by a mixture of two bivariate normal distributions, further supporting bimodality (fig. S5 and table S1; see Materials and Methods for details).

**Fig. 4.**
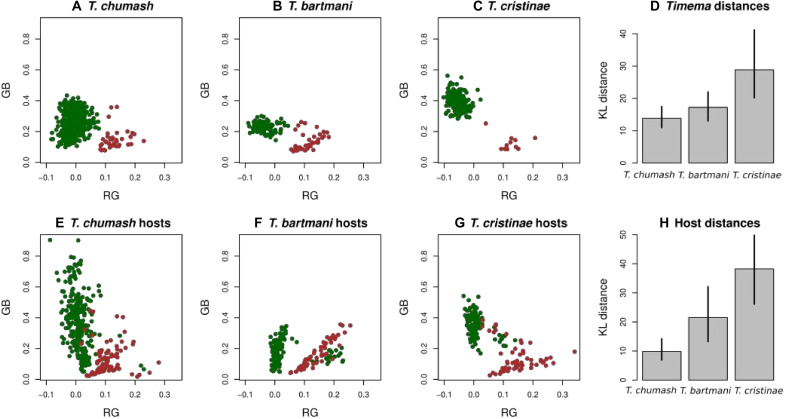
Overlap in coloration between green and melanistic *Timema* morphs, and the leaves versus stems of the host plants they are found upon. For *T. chumash*, this is oak (*Quercus* sp.) and mountain mahogany (*Cercocarpus* sp.); for *T. bartmani*, this is white pine (*Pinus* sp.) and white fir (*A. concolor*); for *T. cristinae*, this is California lilac (*C. spinosus*) and chamise (*A. fasciculatum*). (**A** to **C**) Empirical *Timema* color data, where the green and brown dots are clusters corresponding to green versus melanistic morphs. (**E** to **G**) Plant coloration data. For host plants, the green and brown dots are data from leaves versus stems, respectively. The two right panels (**D** and **H**) show the mean Kullback-Leibler (KL) distance between morphs and host tissues as bars, with vertical lines representing 95% equal-tail probability intervals.

The results above establish the existence of identifiable green and melanistic morphs in all our study species. However, a comparison of the mixture model results, and a visual inspection of the color in the RG and GB color space, suggests that the degree of morph differentiation and bimodality varies (Fig. 4 and fig. S5). We thus quantified morph differentiation among *Timema* species by computing Kullback-Leiber distances between the green and melanistic morphs in each of the three species (see Materials and Methods for details on methods). As suspected, we found that our study species span a range of observed morph differentiation, where the color distance between morphs is *T. chumash* < *T. bartmani* < *T. cristinae* (mean Kullback-Leibler distance between morphs: *T. chumash* = 14.0, *T. bartmani* = 17.4, *T. cristinae* = 29.2; posterior probabilities: *T. bartmani > T. chumash* = 0.87, *T. cristinae* > *T. bartmani* = 0.98, *T. cristinae > T. chumash* ~ 1.0; Fig. 4). These species thus provide the requisite variation to evaluate the ecological discontinuity hypothesis, in our case by comparing morph differentiation to differentiation of the colors of the leaves versus stems of the host plants that they live on.

### Greater color differentiation between leaves and stems is associated with greater differentiation between green and melanistic morphs in *Timema*

We used standardized photographs of 761 host-plant samples to quantify color variation of plant parts (leaves and stems) using methods similar to those applied for *Timema* (see Materials and Methods for details on measurements and methods). Consistent with the ecological discontinuity hypothesis, the hosts of *T. chumash* (*Quercus* sp. and *Cercocarpus* sp.) displayed the widest and most continuous range of variation for coloration in leaves and stems, including shades of blue, green, yellow, tan, beige, brown, and red (Fig. 4). As a result, the hosts of *T. chumash* displayed the lowest color difference between their leaves and stems (mean Kullback-Leibler distance = 10.1; Fig. 4), compared to the hosts of *T. bartmani* (*A. concolor* and *Pinus* sp.; mean Kullback-Leibler distance = 21.8; Fig. 4), and the hosts of *T. cristinae* (*Adenostoma fasciculatum* and *Ceanothus spinosus*; mean Kullback-Leibler distance = 39.3; posterior probabilities, *T. bartmani > T. chumash* hosts = 0.99, *T. cristinae* > *T. bartmani* hosts = 0.97, *T. cristinae > T. chumash* hosts ~1.0; Fig. 4).

The hosts of *T. chumash* may therefore select only weakly against intermediate body coloration, potentially explaining why morphs of *T. chumash* are less discrete. In contrast, the hosts of *T. bartmani* and *T. cristinae* exhibit greater color distance between their leaves and stems than the hosts of *T. chumash*. These hosts thus offer a more bimodal and discontinuous range of colors, primarily green versus brown coloration, which could select more strongly against intermediate body coloration in *T. bartmani* and *T. cristinae* (*30*–*32*).

### Disruptive selection on body coloration in *Timema* is host dependent

Given the preceding observational results, we designed an experiment to explicitly test the prediction of stronger disruptive selection against intermediate body coloration on hosts with greater color discontinuity between their leaves and stems. We tested this prediction using a field-based release-recapture survival study [past work has shown that the recapture probability is a good proxy for survival (*25*, *42*, *43*)]. We did so by marking and transplanting equal numbers of green, melanistic, and intermediately (intermediates, hereafter) colored *T. chumash* to non-natal host plants comprising two treatments: (i) chamise and California lilac (*A. fasciculatum* and *Ceanothus leucodermis*, respectively; these hosts offer highly discrete green and brown coloration; A/C treatment hereafter) and (ii) mountain mahogany (*Cercocarpus* sp.; a host offering more continuous variation in coloration; MM treatment hereafter).

A previous release-recapture experiment involving *T. chumash* and using these two treatments was designed to elucidate which genetic color variants are under selection in natural populations and thus used natural morph frequencies (*25*). By virtue of using natural frequencies, intermediates were rare in this experiment. We thus here designed an experiment better suited to testing the prediction of variation in disruptive selection against intermediates, by enriching the trait distribution for intermediate coloration. Specifically, we collected 602 *T. chumash* individuals as an initial pool, of which 40 (or 6.6%) were intermediates (see Materials and Methods for details). We do not know the frequency of intermediates in the other two species studied observationally above, as they were not studied with this same experimental procedure. However, we note that 6.6% likely represents the upper bound of the frequency of intermediates, as the other two species were found to be more bimodal than *T. chumash*, as reported above.

From this initial pool of 602 *T. chumash* individuals, equal numbers of individuals determined to have green, intermediate, and melanistic coloration were chosen for release and recapture (thus intermediates were enriched from 6.6 to 33.33% in our experiment). In all, a total of 120 insects composed of 40 of each of the three color categories were released into two separate blocks, with each block containing the two plant color treatments MM and A/C (each treatment in a block thus received 30 insects; see Materials and Methods for details). As a result of this design, our current experiment allows more powerful inference of the fitness of intermediates than did past work (*25*). We note that the results reported below are unlikely to be driven by a single predator individual, as we observed multiple birds and lizards foraging in our experimental bushes, both before and during the experiment.

**Fig. 5.**
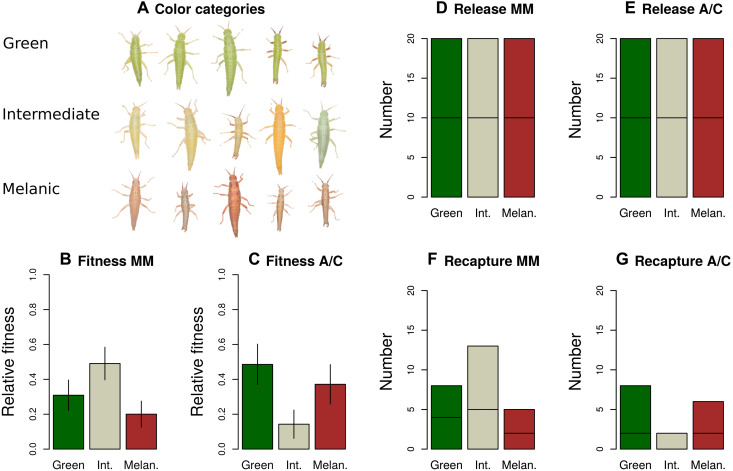
Results of the transplant experiment in *T. chumash* quantifying disruptive selection in different treatments. (**A**) Representatives of the green, melanistic (Melanic), and intermediate color categories in *T. chumash*. (**B** and **C**) Relative fitness of the different categories in each treatment. Bars are means and SDs of the posterior (analogous to SEs). The raw numbers of individuals released and recaptured are shown in (**D** to **G**), where the horizontal line in each bar distinguishes numbers of individuals from each of two experimental blocks.

**Fig. 6.**
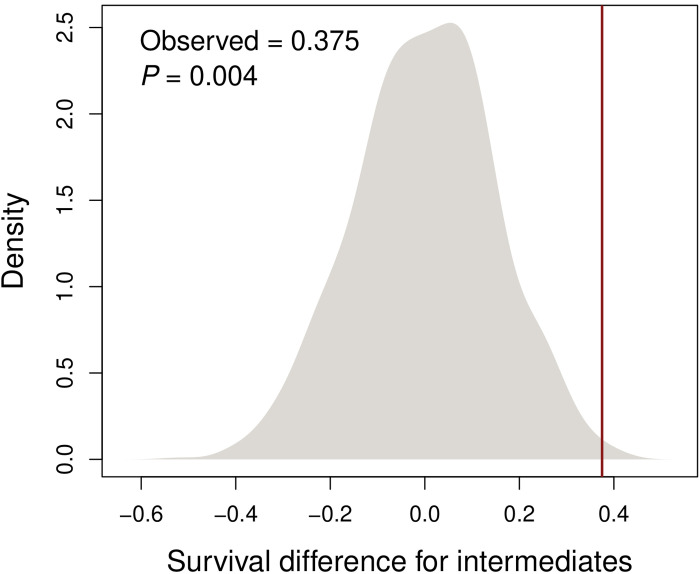
Observed difference in the proportion of recaptures of intermediates in the A/C treatment versus the MM treatment relative to null expectations established via randomization. The density plot shows the expected null distribution from random sampling and also the value that we actually observed in the experiment, the latter shown with a vertical red line.

Consistent with predictions of the discontinuity hypothesis, we recaptured a lower proportion of intermediates in the A/C treatment than in the MM treatment (Fig. 5; posterior probability that survival of intermediates is greater in the MM treatment >0.99, multinomial Dirichlet model). Specifically, we considered three models: (i) a null model where the fitness of intermediates was constrained to be the same in all treatments and blocks, (ii) a model where the fitness of intermediates was allowed to differ between treatments but was constrained to be the same across blocks (no block effect), and (iii) a model where the fitness of intermediates was allowed to differ between treatments and blocks. The model allowing the fitness of intermediates to vary between treatments but not block (i.e., model ii) was preferred by deviance information criterion (DIC; see Table 1). On the basis of this model, we detected strong disruptive selection in the A/C treatment [*s* = −2.73, posterior probability (pp.), that *s* < 0 = 0.97; *t* = −1.84, pp. *t* < 0 = 0.92, where relative fitness is defined as follows: green = 1 − *s*, intermediate = 1, and melanistic = 1 − *t*, i.e., *s* or *t* < 0 implies disruptive selection, and *s* or *t* > 0 implies intermediate advantage]. In contrast, selection in the MM treatment was stabilizing if anything, with intermediates exhibiting the highest survival (*s* = 0.38, pp. *s* < 0 = 0.14; *t* = 0.60, pp. *t* < 0 = 0.03). A nonrandom association between host treatment and survival rates of intermediate morphs was also supported by a 2 × 2 contingency table test (χ^2^ = 4.5433, df = 1, *P* = 0.03305; table S2). Moreover, the observed difference in the recapture rate of intermediates between treatments cannot be explained by sampling error (*P* = 0.004, randomization model; Fig. 6). In summary, the strength of disruptive selection against intermediates in our experiment was host plant dependent, in a manner consistent with the observational results and the discontinuity hypothesis.

**Table 1.** Mean deviance, effective number of parameters, and DIC for three alternative models to explain survival for intermediates in our transplant experiment (i) A null model where the relative fitnesses of intermediates are equal across all treatments and blocks, (ii) a model where the relative fitnesses of intermediates varies across treatments only, and (iii) a model where the relative fitnesses of intermediates varies across treatments and blocks. pD, effective number of parameters.

Model	Mean deviance	pD	DIC
Null (i)	28.22	1.92	30.14
Treatment (ii)	23.38	3.73	27.12
Treatment-by-block (iii)	25.25	6.88	32.14

### Morph differentiation has different genetic consequences in *Timema*

Last, we tested the genetic consequences of morph differentiation because this might potentially have implications for adaptation and reproductive isolation. To this end, we used the two *Timema* species (i.e., *T. bartmani* and *T. cristinae*) whose body color is associated with a single region of reduced recombination (a complex structural variant including inversions and deletions, the *Mel-Stripe* locus) (*26*) such that we could readily study genotype frequencies at this locus. Specifically, we estimated the difference between expected (under Hardy-Weinberg equilibrium) and observed heterozygosity of the *Mel-Stripe* locus in these two species.

**Fig. 7.**
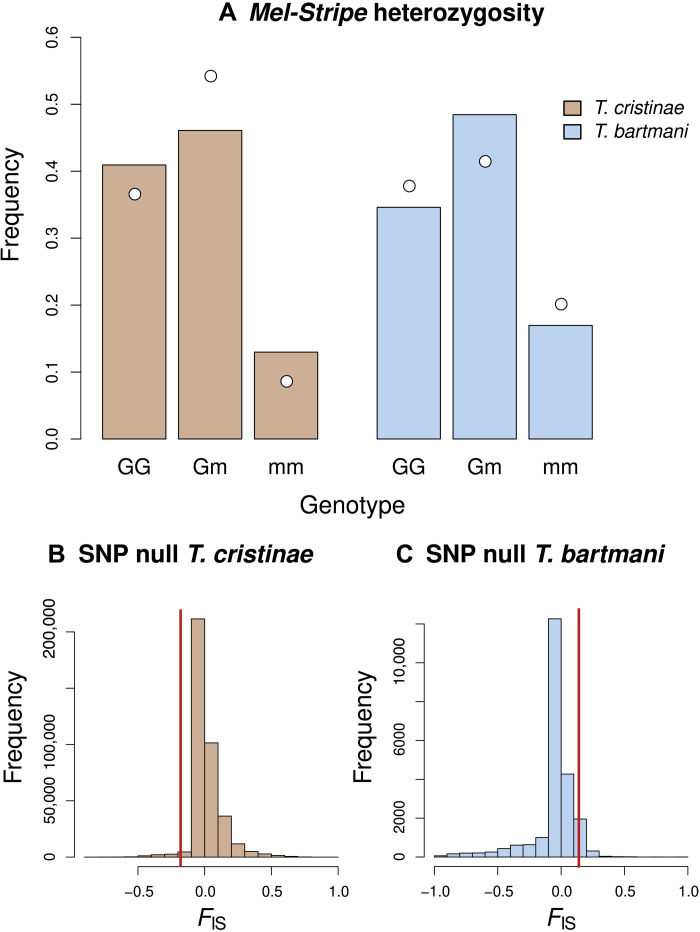
Observed and expected heterozygosity at the *Mel-Stripe* locus in *T. bartmani* and *T. cristinae* and comparison with genome-wide *F*_IS_. (**A**) *Mel-Stripe* heterozygosity. *Mel-Stripe* genotypes (GG = homozygous green, Gm = heterozygous, mm = homozygous melanistic) were inferred from principal components analysis and *k*-means clustering of genetic variation within *Mel-Stripe* (see Materials and Methods for details). Colored bars denote expected *Mel-Stripe* genotype frequencies under Hardy-Weinberg equilibrium, and white dots denote the corresponding observed genotype frequencies. For each species, a single population was used [*T. bartmani*: Jenks Lake (JL) population, 408 individuals; *T. cristinae*: Far Hill *Adenostoma* (FHA) population, 508 individuals]. (**B** and **C**) Comparison of *Mel-Stripe F*_IS_ and genome-wide *F*_IS_. The distribution of *F*_IS_ for genome-wide SNPs is shown as a histogram for each species and the *F*_IS_ for *Mel-Stripe* is denoted by vertical red lines.

We found a significant deficiency of heterozygotes in *T. bartmani* (observed heterozygosity = 0.42, expected heterozygosity under Hardy-Weinberg equilibrium = 0.48, χ^2^ = 7.98, *P =* 0.0181; Fig. 7). Such a deficiency of heterozygotes hints at potential reproductive barriers between morphs of this species (see Discussion for possible causes, of which natural selection is one). The result in *T. bartmani* is in stark contrast to *T. cristinae*, where we detected an excess of heterozygotes (observed heterozygosity = 0.54, expected heterozygosity under Hardy-Weinberg equilibrium = 0.46, χ^2^ = 19.21, *P =* 0.0001; Fig. 7A), as reported in previous work (*44*).

Moreover, the excess of heterozygosity at the *Mel-Stripe* locus in *T. cristinae* exceeded null expectations based on the genome-wide distribution of heterozygosity for 382,118 single-nucleotide polymorphisms (SNPs) (*Mel-Stripe F*_IS_ = −0.180 versus genome-wide mean *F*_IS_ = 0.024, first quartile = −0.023, third quartile = 0.055, one-tailed *P* = 0.0180). The deficit of heterozygosity at the *Mel-Stripe* locus in *T. bartmani* was likewise marginally (but not significantly) more extreme than expected from the genome-wide distribution of heterozygosity in 22,827 SNPs (*Mel-Stripe F*_IS_ = 0.140 versus genome-wide mean *F*_IS_ = −0.051, first quartile = −0.053, third quartile = 0.019, one-tailed *P* = 0.0583) (Fig. 7, B and C). In summary, these results support variable consequences of morph differentiation for genetic differentiation at the *Mel-stripe* locus in *Timema* stick insects.

## DISCUSSION

We studied how variation in ecological discontinuity and disruptive selection is associated with variation in the discreteness of phenotypic forms. Specifically, we showed that greater differentiation between the leaves and stems color of host plants is associated with greater differentiation between green and melanistic morphs in *Timema* stick insects. We hypothesized that the mechanism behind this observation is stronger disruptive selection on plants with higher discontinuity for coloration and designed an experiment that tested and supported this idea. Our experiment shows that, at least in part, host plant coloration causally affects selection on *Timema* coloration and thus the level of discontinuity of this trait.

Our work adds to other major studies on the evolution of divergent phenotypes and their maintenance via ecologically based disruptive selection. Examples of such ecologically based disruptive selection include the following: *Littorina* intertidal snails where substrate heterogeneity affects coloration (*2*); *Rhagoletis* flies were host preference selects for different diapause time (*45*); the black-bellied seedcracker (*Pyrenestes ostrinus*) and Darwin’s finches (*Geospiza* sp.) where intra- and interspecific competition during dry periods interact with seed availability and select for different beak sizes (*14*, *46*); the three-spined stickleback (*Gasterosteus aculeatus*) where differences in predator regime likely select for differences in bony armor (*16*, *47*, *48*); and deer mice (*Peromyscus* sp.), where avian predation interacts with soil coloration and likely selects for different coat coloration (*15*, *49*). Our work, however, goes beyond documenting disruptive selection to show that different degrees of discontinuity for an ecological variable affect the level of discreteness of the phenotype it selects (in our case coloration).

Our results also show how ecological discontinuities can be associated with variation in the genetic architecture of traits. In our study, the *Timema* species found on host plants with the strongest color differentiation between leaves and stems (*T. bartmani* and *T. cristinae*) showed the strongest differentiation between green and melanistic morphs. In these two species, body coloration is associated with a region of reduced recombination on LG8 (i.e., the *Mel-Stripe* locus, where recombination is very strongly reduced) (*26*) and strong dominance (i.e., the green allele is dominant over the melanistic allele) (*19*, *24*, *26*). In contrast, the species found on host plants with the lowest differentiation between leaves and stems (*T. chumash*) shows less differentiation between green and melanistic morphs. In this species, color also maps to the *Mel-Stripe* locus but is associated with five normally recombining loci (located near several candidate genes) in this region (*25*, *26*). It is therefore likely that these multiple recombining loci in *T. chumash* exist and segregate as a single genetic unit in *T. barmani* and *T. cristinae* (*26*). Such a genetic unit is a predicted theoretical outcome when disruptive selection acts on multiple loci located on the same chromosome (*50*–*53*). If a rearrangement suppressing or strongly reducing recombination (like an inversion or a translocation) arises and locks together a favorable allelic combination at these loci, then this rearrangement can be selected for because it prevents the creation of less fit genotypes generated by recombination (*52*). This new rearrangement will segregate as a single genetic unit (i.e., a supergene). Thus, in *Timema*, color differentiation between leaves and stems, by virtue of affecting disruptive selection, could potentially help explain the variability in genetic architecture for body coloration in *Timema* and the evolution of putative supergenes (*26*, *29*, *54*). In the same fashion, dominance of the green alleles could have evolved to further reduce the production of intermediate color phenotypes, as may occur for dominance evolution of mimetic coloration in *Heliconius* butterflies (*55*). An open question in *Timema* is the timing of changes in selective regime (e.g., via a host shift) relative to the age of the rearrangements associated with body coloration. Did the structural genomic variation precede host shifts (and may have facilitated it in some ways), or did it arise after a host shift and spread because of host plant–induced disruptive selection? This issue remains an open question in *Timema* but also in most other putative supergene systems and warrants further attention (*54*).

Last, we quantified the consequence of morph differentiation for genetic differentiation at the locus controlling body coloration (i.e., the *Mel-Stripe* locus). We found evidence for heterozygote deficiency at the *Mel-Stripe* locus in *T. bartmani.* This deficiency is marginally (but not significantly) stronger than deficiencies observed in the rest of the genome. Nonetheless, this suggests that color morphs in this species may not be entirely freely interbreeding (in the broad sense of selection on viability, mating, and dispersal, not restricted to mating preferences per se). This pattern could arise from prezygotic barriers to reproduction, such as assortative mating between color morphs, or microhabitat segregation between morphs. However, there is currently no evidence in *Timema* supporting either assortative mating according to morph (*19*) or microhabitat segregation between morphs. Moreover, we did not detect strong and general departure from Hardy-Weinberg expectations at the genome-wide level (Fig. 7), and green and melanistic morphs are often seen intermating in natural populations (*56*). A deficiency of heterozygotes could also come from postzygotic barriers to reproduction, such as ecologically based selection against intermediate phenotypes (likely here on other traits than body coloration, due to the aforementioned genetic dominance of color) or intrinsic genetic incompatibilities. Because our results suggest that selection could act against intermediate body coloration in *T. bartmani*, and because genetic dominance is strong for body coloration in this species, selection against heterozygotes would need to involve coloration differences that we did not measure or pleiotropic effects of the *Mel-Strip*e locus on traits other than coloration. In contrast to *T. bartmani*, we confirmed past evidence for an excess of heterozygotes in *T. cristinae* (*44*). Although the causes of this pattern remain unclear, past work does support heterozygote advantage (*20*, *44*) and argues against disassortative mating (*19*). Collectively, our results suggest potentially variable consequences of phenotypic differentiation for genetic differentiation at the *Mel-Stripe* locus.

In conclusion, we here report a role for ecological discontinuities in the evolution of discrete forms (i.e., color morphs). Such discontinuity may thus help reconcile large evolutionary shifts (i.e., ideas concerning macromutation and “hopeful monsters”) (*57*–*59*) with polygenic adaptation and neo-Darwinian gradualism (*7*, *60*, *61*). Specifically, the plausibility and mechanisms of large or sudden evolutionary changes remain unclear (*62*–*64*). Developmental biology provides one possible mechanism: developmental switches involving gene regulation (*62*–*64*). Our results illustrate another: the conversion of genetic variation into discrete phenotypic units via strong disruptive selection associated with ecological discontinuities, and perhaps facilitated by supergene evolution.

## MATERIALS AND METHODS

### *Timema* sampling

Following past work, we collected *Timema* by shaking the branches of host plants while holding a sweep net underneath them (*19*). We stored adult (i.e., sexually mature) specimens in plastic containers for immediate phenotyping (reflectance analysis or photographing; see sections below). Following past work, we reared juvenile individuals on *C. spinosus* cuttings in plastic containers until they reached adulthood (with the exception of *T. bartmani* individuals, which were analyzed as juveniles; see details in sections below) (*19*) and then phenotyped them. We stored each photographed specimen in an individual vial with pure ethanol for subsequent molecular work.

### Reflectance data and spectrophotometry of *Timema* and their host plants

We quantified ultraviolet and other spectral reflectance in *Timema* and their host plants. To do so, we acquired new spectral reflectance data for *T. bartmani* individuals and reanalyzed spectral reflectance data of *T. cristinae* and *T. chumash* (table S3) (*19*, *25*). For *T. cristinae*, we randomly sampled five individuals from each morph (i.e., five green individuals and five brown individuals) from past work (*19*, *25*). For host plants, we acquired new spectral reflectance data for *Cercocarpus* sp. and *A. concolor* and reanalyzed data from past work (*19*) for *A. fasciculatum* and *C. spinosus* (table S4).

We recorded spectral data as detailed in (*25*). Briefly, we collected spectral data using a USB2000+ Fibre Optic Spectrometer (Ocean Optics Inc.) equipped with a 400-μm reflection probe (R400-7-SR) and a pulsed xenon lamp (PX-2) with an output spectrum of 220 to 750 nm. We measured reflectance at a 45° angle with an integration time of 50 ms, with the boxcar width adjusted to 5, and averaged measures across 20 scans. We then corrected these measurements for nonlinearity, stray light, and electric dark using the OceanView software (Ocean Optics). We measured reflectance relative to a Spectralon >99% white standard provided by the manufacturer (WS-1).

We euthanized *Timema* individuals just before measuring reflectance using a killing jar filled with acetone. For each *Timema* individual, we recorded two reflectance spectra, one from the dorsal anterior part of the body (comprising thorax and head) and another from the dorsal posterior part (abdomen). We interpolated raw reflectance between 300 and 700 nm, corrected negative values to zero, and applied triangular smoothing with a distance of 10 nm using the software AVICOL (*65*). Last, we estimated mean reflectance as the averaged reflectance between the dorsal and abdominal measurements. We then estimated the mean reflectance across the measured spectrum (220 to 750 nm) for each morph of each species.

For each leaf sample, we acquired two reflectance spectra: one from the upper part of the leaf (i.e., adaxial) and another from the lower part of the leaf (i.e., abaxial). For *A. fasciculatum*, past studies recorded only one reflectance spectrum per leaf sample because their needle-like shape prevented the identification of an upper and lower side (*19*). For stems, we recorded one reflectance spectrum per sample as the concept of upper and lower surface does not apply. We processed the raw data as described above using AVICOL (*65*). When available, we pooled upper and lower reflectance spectra of leaves to contrast them with the reflectance spectra of stems.

### Birds’ color perception of *Timema* and their host plants

We estimated birds’ perception of the coloration of *Timema* and host plants with a quantum catch analysis. Specifically, this analysis estimates how bird color cones are stimulated by a given coloration signal. We followed a previously published pipeline for this quantum catch measurement (*26*). Given most birds predating on *Timema* belong to the Passerine order, we calculated quantum catch estimates using Passerine photoreceptors’ sensitivities (i.e., the average ultraviolet sensitive system) (*66*, *67*). We performed one quantum catch analysis for each morph across *Timema* species (i.e., one for the green morph and for the melanistic morph), pooling values from green or melanistic individuals across *Timema*’s species. To do so, we averaged reflectance at each nanometer for all individuals of a given morph. We conducted these analyses using the R package pavo (*38*). We used the same method for host plants, averaging reflectance at each nanometer across samples of the same tissue (i.e., leaves or stems) and species type.

### Measurements of *Timema* coloration from photographs

We reanalyzed body color data from (*26*) (table S5). A more detailed description of our methods is available in (*26*), but we provide a brief description of methods below.

To measure *Timema* body color, we took standardized digital photographs of adult individuals for all species but *T. bartmani* that develop later in the season. For *T. bartmani*, we photographed juveniles. Note that our core conclusions are unaffected by this as they do not rely on *T. bartmani* alone (e.g., the experiment described below does not rely on *T. bartmani*), and current and past work shows that color morph is highly heritable such that it persists across life-history stages (*19*, *20*, *24*–*26*, *44*).

We photographed all individuals with a digital Canon EOS 70D camera equipped with a macro lens (Canon EF 100 mm f/2.8 L Macro IS USM) and two external flashes (Yongnuo YN560-II speedlights). We set the camera on manual, an aperture of f/14, a shutter speed of 1/250 s, and a sensitivity of 100 ISO. We set two external flashes to provide standardized light and adjusted them to one-fourth power in S2 mode and in an output angle corresponding to 24-mm focal length on full frame (~84° diagonal). We attached LumiQuest SoftBox LTp softboxes to the flashes to produce a more homogeneous light and avoid gleam. In addition to the *Timema* individuals, we included a ruler and a standard color chip in the photograph frame (Colorgauge Micro, Image Science Associates LLC, Williamson, NY, USA).

We photographed each individual in different perpendicular positions to capture body coloration while minimizing gleam or shade. We linearized photographs and corrected them for white balance using Adobe Photoshop Lightroom 5.7 software (Adobe Systems Software Ireland Ltd), adjusting temperature and the tint 1 on the values obtained from the color chip neutral gray color (target #10). We adjusted the photographs for a temperature of 5950 and for a tint to +2 and exported them as TIFF files.

We measured body coloration from the standardized photographs using the software ImageJ 1.4.882 (*68*). Specifically, we recorded mean RGB values using the polygon section tool and color histogram plugin. For every *Timema* individual, we measured a small area in the lateral region of the insect’s thorax and abdomen.

Following (*41*), we measured the relative difference between the red and green color channels (RG hereafter) and between the green and blue color channels (GB hereafter) for a better interpretation of the RGB values (Fig. 2). We computed the RG and GB traits (*41*) as follows

$${RG}_{i}=(R_{i}-G_{i})/(R_{i}+G_{i})$$ (1)

$${GB}_{i}=(G_{i}-B_{i})/(G_{i}+B_{i})$$ (2)

where B_i_ is the mean value of individual i in the blue color channel, G_i_ is the mean value of individual i in the green color channel, and R_i_ is the mean value of individual i in the red color channel.

Although this method does not take into consideration how color is sensed by predators, it does yield a standardized and replicable quantification of color that can be used in a comparative framework. Similar procedures in past work showed that our coloration measurements are highly repeatable (*19*, *43*, *69*).

### Differentiation and overlap between *Timema* green and melanistic morphs

We used the UPGMA algorithm in the R package hclust to cluster *Timema* into two groups (i.e., color morphs) based on the RG and GB traits. Morphs were defined on the basis of assignment to these clusters.

Next, as an independent and complementary analysis, we fit mixture models to the RG and GB estimates to determine whether the color data were better explained by one or two (i.e., a mixture of) distributions, as expected for unimodal versus bimodal color distributions, respectively. We use the flexmix function and package (version 2.3.18) in R (versions 4.1.3) to fit these models (*70*). For each species, we fit bivariate normal models with one versus two distributions using an expectation-maximization algorithm. We set the tolerance to 1 × 10^−15^ and the maximum number of iterations to 1000. We compared models using Akaike information criterion (AIC), with lower AIC values indicating better fit.

Next, to quantify color divergence between morphs, we used a Bayesian approach to fitting RG and GB traits for each morph (as defined by hierarchical clustering) to a bivariate normal distribution. We placed relatively uninformative priors on the mean vectors (normal with µ = 0 and τ = 1 × 10^−3^ for both means) and for the precision matrix (Wishart with two degrees of freedom and a diagonal scale matrix = 0.001 ***I***, where ***I*** is the identity matrix). We used Markov chain Monte Carlo (MCMC) to obtain samples from the posterior distribution via the rjags (version 4.6) interface with JAGS (version 4.1.0) (1000 iteration burn-in, 5000 sampling iterations, and a thinning interval of 4). We then estimated the Kullback-Leibler distances (i.e., the Kullback-Leibler divergence in both directions, from green morph to melanistic morph and melanistic morph to green morph). We calculated this distance over the posterior distribution of the bivariate normal parameters, and it thus accounts for uncertainty in these parameters.

### Phenotypic measurements of leaves and stems coloration from photographs

We quantified the coloration of the host plants of *T. chumash*, *T. bartmani*, and *T. cristinae* using a photographic procedure similar to that described for *Timema* individuals. We collected plant cuttings (table S6) and kept them in a cooler for a maximum of a few days until we photographed them.

We applied the same standardization procedures as described above for *Timema* individuals. We took multiple measurements in each photograph, each counting as a plant sample. We measured an average of 3.8 samples per photograph (3.4 to 4.1; 95% confidence interval). To estimate the RG and GB traits for leaves and stems, we measured RGB values in a circular area of 1-mm diameter. This allowed us to measure samples of broad leaves and individual needles using the same surface area. For plants species with broad leaves (i.e., *Ceanothus* sp., *Cercocarpus* sp., and *Quercus* sp.), we recorded the RGB values for the upper (adaxial) and lower (abaxial) leaf surfaces and computed the RG and GB values from the upper and lower surfaces independently. This was done to comprise a larger diversity of colors in host plants. For plants with needle-like leaves (i.e., *A. concolor*, *A. fasciculatum*, and *Pinus* sp.), we recorded one measurement if the surface was uniform in color (i.e., *A. fasciculatum* and *Pinus* sp.) or two if color varied between the upper and lower surfaces (i.e., *A. concolor*). In the latter case, we computed the RG and GB values for the upper and lower surfaces independently. Measurements from the upper and lower surfaces of the leaves were grouped together as “leaf” in the downstream analyses (see below).

### Differentiation and overlap in coloration between leaves and stems

We estimated Kullback-Leibler distances between leaves and stems using a method similar to that we described for *Timema* body coloration (see the “Differentiation and overlap between *Timema* green and melanistic morphs” section).

### Manipulative field experiment for quantifying disruptive selection

We estimated selection on color by marking and transplanting green, melanistic, and intermediately colored *T. chumash* to two treatments: (i) hosts associated with highly discrete morphs (chamise and California lilac, *Adenostoma* *fasciculatum* and *Ceanothus* *luciferum* respectively, A/C treatment) versus (ii) a host associated with less discrete morphs (mountain mahogany, *Cercocarpus* sp., MM treatment). We used *T. chumash* because this species exhibits the most continuous range of color such that reasonable numbers of “intermediately” colored individuals can be collected to have their survival assayed (alongside with clearly green or melanistic individuals).

We collected the experimental *T. chumash* from *Cercocarpus* sp. in the vicinity of the locality Horse Flats 5 (HF5; N 34° 15.584′, W 118° 6.254′). We collected a total of 602 individuals between 9 May and 11 May 2018. We kept them alive in plastic deli cups and moved them to laboratory space on the campus of University of California, Santa Barbara, USA.

On 12 May 2018, we assigned 120 of these individuals into three color categories: greens, intermediates, and melanistics. To represent the green category, we selected 40 of the brightest and darkest green specimens. To represent the intermediate category, we selected 40 individuals with green-yellow, yellow, brown-yellow, and green-blue tones, as these collectively depict the transition from green to melanistic morphs. For this category, green-yellow coloration was present in the majority of individuals. These 40 individuals represent all the intermediates that we captured such that the overall intermediate frequency can be estimated as 40 of 602 = 6.6%, with enrichment in the experiment to 40 of 120 = 33.3%. To represent the melanistic category, we selected 40 individuals with the darkest brown and red coloration.

We estimated the repeatability of this scoring to be 96% (95% equal-tail probability intervals from a Bayesian beta-binomial model with a Jeffreys prior = 88 to 99%, this model has an analytical solution) by scoring 50 individuals twice, where only two scoring errors were made. Specifically, 21 individuals were scored as green both times, 15 individuals were scored as intermediate both times, 12 individuals were scored as melanistic both times, and 2 individuals were scored as intermediate and green once. Representative specimens of each category are shown in Fig. 5.

Following past work (*21*, *43*), we marked each individual on the abdomen with a fine-tipped sharpie pen to ensure that we could distinguish our experimental animals from naturally occurring ones at recapture. The marks were thus not visible when the insects were naturally resting on their host plants. Each color category (greens, intermediates, and melanistics) received a differently colored mark, facilitating accurate rescoring of color in recaptured specimens. As our experimental design involved two blocks (details below), we alternated which color mark was assigned to which category (block 1: greens marked with a blue pen, intermediates marked with a green pen, and melanistics marked with a red pen; block 2: greens marked with a green pen, intermediates marked with a red pen, and melanistics marked with a blue pen).

On 13 May 2018, we transplanted the marked specimens back onto host plants at the locality we originally collected them from. We designed an experiment with two blocks, where each block contained both plant treatments (MM and A/C), using a single plant for transplant of each host species. We released equal numbers of greens, intermediates, and melanistics on each of the two treatments within each of the two block (i.e., 10 individuals of each color category in each treatment and block, total *n* = 120 for the experiment). The location of each experimental plant was as follows: block 1: MM N 34° 15.584′, W 118° 6.254′, A/C N 34° 15.599′ W 118° 6.256′; block 2: MM N 34° 15.682′ W 118° 6.127′, A/C N 34° 15.631′ W 118° 6.216′). We chose experimental plants that were separated from other plants by “bare ground” (sandy or gravelly regions not containing plants), forming an “experimental island.” Past work has shown that dispersal across such bare ground is near absent (*42*, *43*, *71*–*73*).

We were interested in rapid changes in the frequency of each color category because past studies in *Timema* have documented adaptive divergence between experimental populations within a week upon transplantation to new environments, and because adult and penultimate instar *Timema* tend to live for only 1 to 3 weeks in the field, with bird predation being a major source of selective mortality (*42*, *43*, *71*, *72*). Thus, on 15 May 2018, we recaptured the surviving individuals using sweep nets and visual surveys. In total, the number of recaptured individuals of each category and treatment was as follows (see also Fig. 7). On MM, we recaptured 8 greens, 13 intermediates, and 5 melanistics. On A/C, we recaptured 8 greens, 2 intermediates, and 6 melanistics. Past mark-recapture work has shown that this protocol is highly effective at recapturing the overwhelming majority of surviving individuals (*42*, *43*, *71*–*73*).

### Null distribution and test for higher survival of intermediates in the MM than in the A/C treatment

We used three complementary approaches to test the null hypothesis of equal relative fitness of intermediates between the two treatments (i.e., MM and A/C). First, we randomly sampled from binomial distributions where *n* was the total number recaptured for a treatment and block and *p* was ^1^/_3_ (the release frequency of intermediates). This allowed us to determine the difference in recapture frequencies between treatments expected by chance while accounting for the specific number of stick insects released and recaptured. We repeated this procedure 1000 times to generate a null distribution for the difference in relative fitness of intermediates between treatments (i.e., the proportion of recaptures that were intermediates in each treatment across blocks), which was compared to the observed value.

Second, we conducted a two-sample proportion test of the null hypothesis that the proportion of recaptures that were intermediates was the same across treatments.

Third, using the chiseq.test function in R (*74*), we conducted a 2 × 2 contingency table test to evaluate the null hypothesis that the relative survival of intermediates (versus nonintermediates: greens plus melanistics) was independent of host treatment (table S2). We used the Yate’s continuity correction for the 2 × 2 contingency table.

### Test for disruptive selection in our transplant experiment

To further analyze the data and explicitly estimate selection, we fit a Bayesian multinomial Dirichlet model to these data using the rjags interface with JAGS (JAGS version 4.1.0, rjags version 4.6, R version 3.2.3). Specifically, recapture counts were assumed to follow a multinomial distribution with a vector *w* of length three, which gives the relative fitness of the three color categories. These relative fitness can be rescaled (e.g., relative to the fitness of any one color category) to aid interpretation of the results. We placed an uninformative Jeffreys Dirichlet prior on this vector (all shape parameters set to 0.5). We considered three models, one where treatments and blocks had the same *w* vectors (model i; “Null” model), one where treatments had independent *w* vectors but blocks had the same *w* vectors (model ii; “Treatment” model), and one where treatments and blocks all had independent *w* vectors (model iii; “Treatment-by-block” model). Posterior distributions were obtained by running three MCMC chains each with a 1000 iteration burn-in, 9000 sampling iterations, and thinning intervals of 3. The Treatment model was preferred by DIC (DIC = 27.12 for the Treatment model versus 30.14 and 32.14 for the Null and Treatment-by-block models, respectively; Table 1), and thus we focus on results from that model. We defined the relative fitnesses (*w*) of the color categories as *w*_greens_ = 1 − *s*, *w*_intermediates_ = 1, and *w*_melanistics_ = 1 − *t* [as in equation 1.25c in (*75*)].

### Departure from Hardy-Weinberg equilibrium at the *Mel-Stripe* locus

We tested for an excess or deficit of heterozygotes (relative to expected heterozygosity under Hardy-Weinberg equilibrium) at the *Mel-Stripe* locus in *T. bartmani* and *T. cristinae*. This locus spans ~10 megabase pairs on linkage group (LG) 8 of the *T. cristinae* genome (*20*, *26*). This region exhibits very limited recombination in *T. bartmani* and *T. cristinae* and likely constitutes a large inversion and/or more complex structural variation (*26*). Thus, as in past work, we assigned individuals *Mel-Stripe* genotypes based on principal components analysis (PCA) and *k*-means clustering of genetic variation within this locus (*44*). This resulted in clear, well-defined clusters of individuals in PCA space.

Here, we used previously published genotyping-by-sequencing data and corresponding genotype estimates from *T. bartmani* [408 stick insects from the Jenks Lake (JL) population, 22,827 SNPs] and *T. cristinae* [590 stick insects from the Far Hill *Adenostoma* (FHA) population, 382,118 SNPs] to estimate *Mel-Stripe* genotypes [see (*26*) for details on these genetic data]. We first used PCA to summarize genetic variation within *Mel-Stripe* for each of these species. This was done in R with the prcomp function and using the centered and scaled genotype matrix. Then, as in (*44*), we used *k*-means clustering with *k* = 6 to assign individuals to LG8 genotypes based on their PC scores for PCs 1 and 2 (mm = homozygous melanistic, Gm = heterozygote for the melanistic allele and the green or green-striped allele, and GG = homozygous for the green or green-striped alleles). We implemented this in R with the *k*-means function with 100 maximum iterations and 100 starts.

Next, we computed observed heterozygosity for the *Mel-Stripe* locus directly from the *k*-means cluster assignments and computed expected heterozygosity under Hardy-Weinberg equilibrium from the corresponding *Mel-Stripe* allele frequencies. We used a chi-squared goodness-of-fit test to assess the statistical significance of deviations between observed and expected heterozygote frequencies. We implemented this with the chisq.test function in R (*74*).

Last, we asked whether the documented excess or deficit of heterozygosity for the *Mel-Stripe* locus was more extreme than expected by chance given the rest of the genome. For this, we first computed *F*_IS_ for *Mel-Stripe* in each species as (HE-HO)/HE where HE and HO are the expected and observed heterozygosities. We then similarly computed *F*_IS_ for each of the 22,827 or 382,118 genome-wide SNPs in *T. bartmani* and *T. cristinae*, respectively. We rounded our Bayesian estimates of genotypes (which were not constrained to integer values) to the nearest integer for this and thus counted heterozygotes as individuals with a value of 1 for each SNP. We then computed the *P* value for *Mel-Stripe* having a more extreme *F*_IS_ value than expected based on the genome-wide SNP distribution by determining its quantile within this distribution.

### Ethical treatment of insects used in this study

While no ethic committee approbation is necessary in France or the United States for experimentation on insects, we handled all individuals with the utmost care to avoid and minimize their suffering. We also used a reasonable number of individuals in all our analyses and experiment to detect small biological effects.
