# Supplementary material for: Disruptive selection and the evolution of discrete color morphs in Timema stick insects
Source: Sci Adv. 2023 Mar 31;9(13):eabm8157. doi: 10.1126/sciadv.abm8157 (PMC10065444; doi:10.1126/sciadv.abm8157)
Supplement: Supplementary file 1 — Figs. S1 to S5 Tables S1 to S6 [file sciadv.abm8157_sm.pdf]

Science Advances
AAAS

Supplementary Materials for

Disruptive selection and the evolution of discrete color morphs in *Timema* stick insects

Romain Villoutreix *et al.*

Corresponding author: Romain Villoutreix, romain.villoutreix@gmail.com; Patrik Nosil, patrik.nosil@cefe.cnrs.fr

Sci. Adv. **9**, eabm8157 (2023)
DOI: 10.1126/sciadv.abm8157

This PDF file includes:

Figs. S1 to S5
Tables S1 to S6

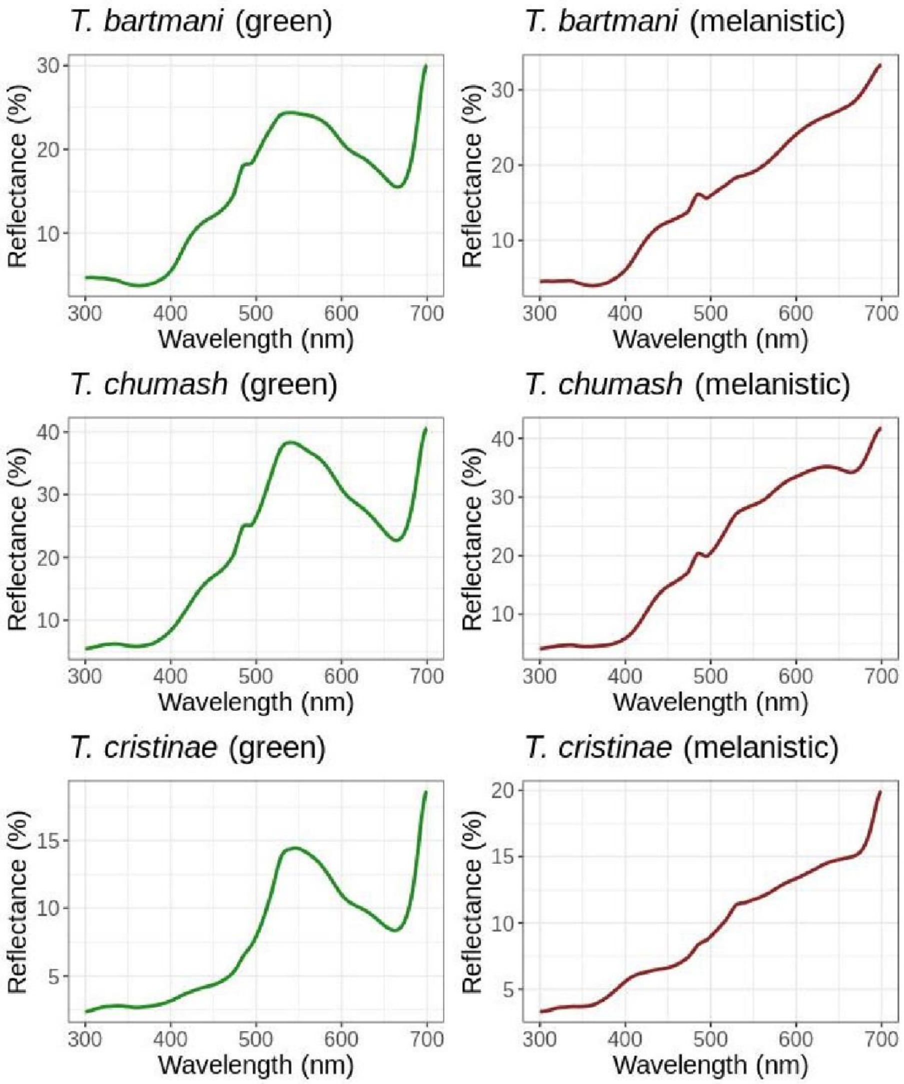

Fig. S1. Reflectance curves for green and melanistic *Timema* morphs.

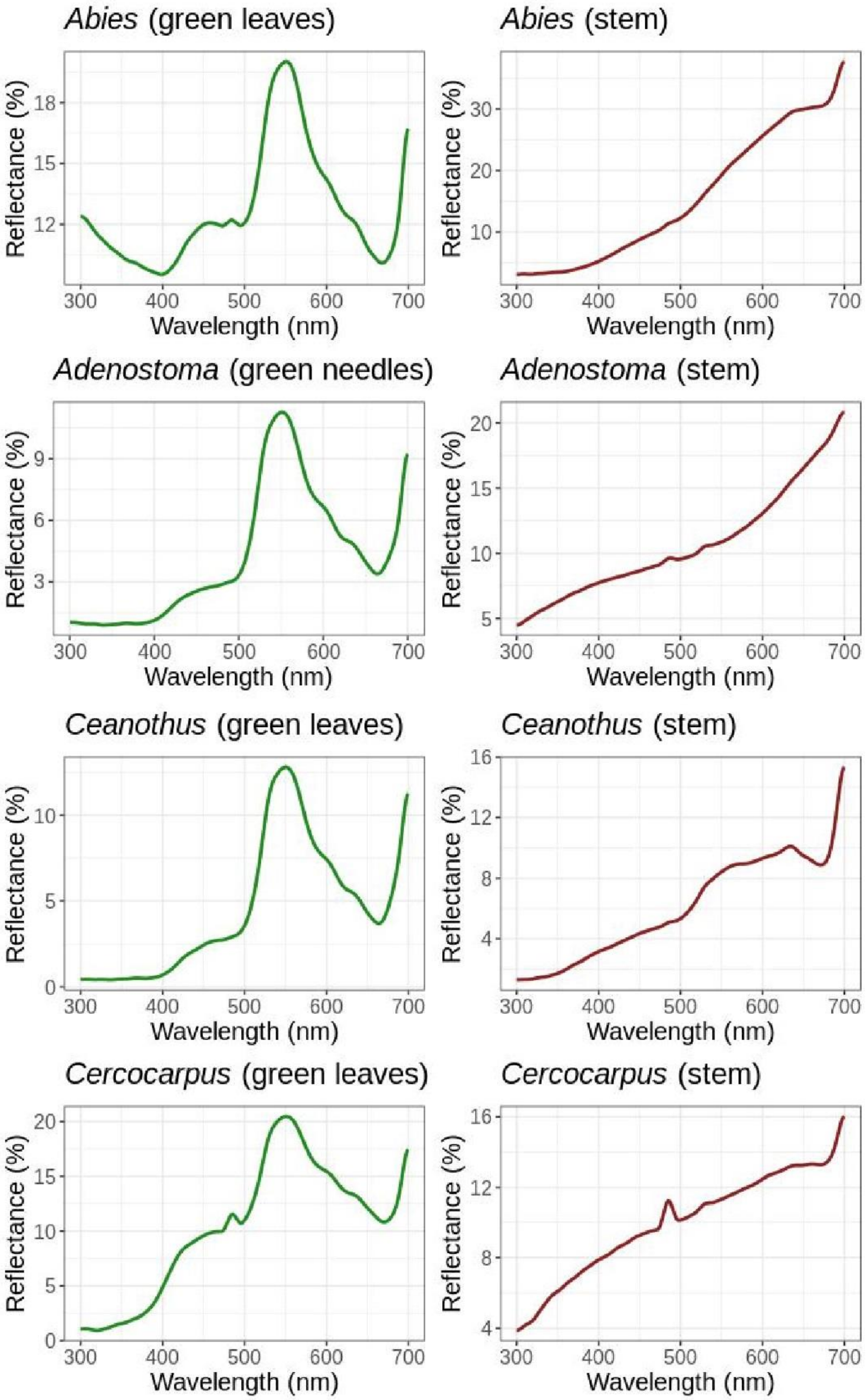

Fig. S2. Reflectance curves for leaves and stems of different host plants.

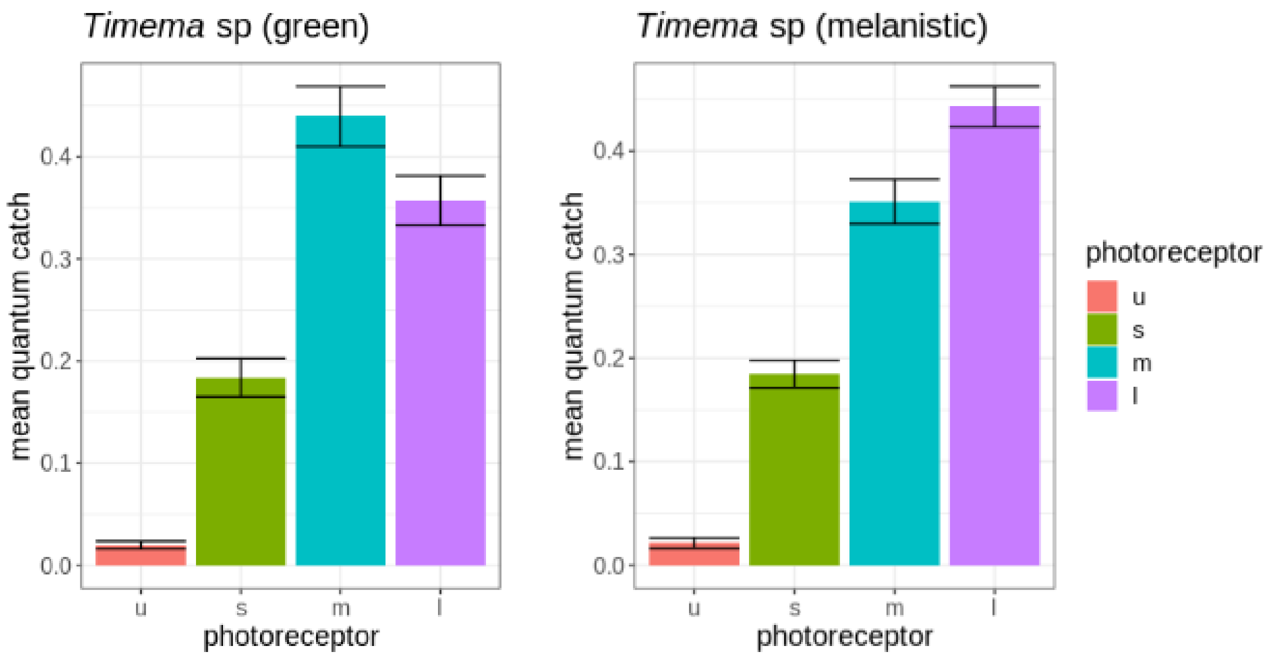

Fig. S3. Quantum catch analysis for green and melanistic *Timema* morphs.

Green and brown morphs of the three *Timema* species were analyzed together (one analysis per morph), with their reflectance values averaged at each nanometer. u = ultraviolet photoreceptor (ultraviolet light), s = small wavelength photoreceptor (blue light), m = medium wavelength photoreceptor (green light), and l = long wavelength photoreceptor (red light).

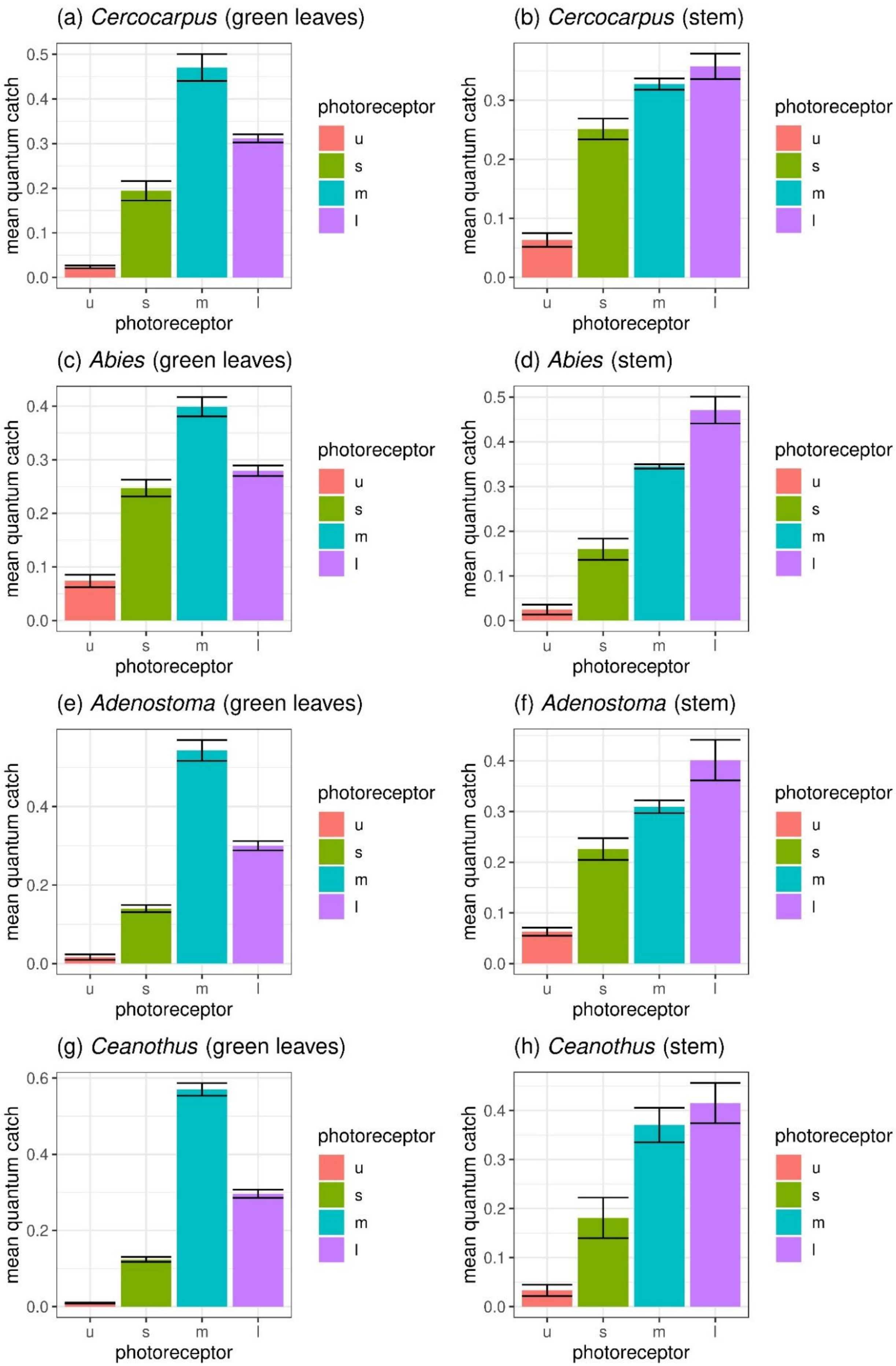
(a) Cercocarpus (green leaves)
(b) Cercocarpus (stem)
(c) Abies (green leaves)
(d) Abies (stem)
(e) Adenostoma (green leaves)
(f) Adenostoma (stem)
(g) Ceanothus (green leaves)
(h) Ceanothus (stem)
mean quantum catch
photoreceptor
u
s
m
l
0.0
0.1
0.2
0.3
0.4
0.5
0.6

Fig. S4. Quantum catch analysis for leaves and stems of different host plants.

u = ultraviolet photoreceptor (ultraviolet light), s = small wavelength photoreceptor (blue light), m = medium wavelength photoreceptor (green light), and l = long wavelength photoreceptor (red light).

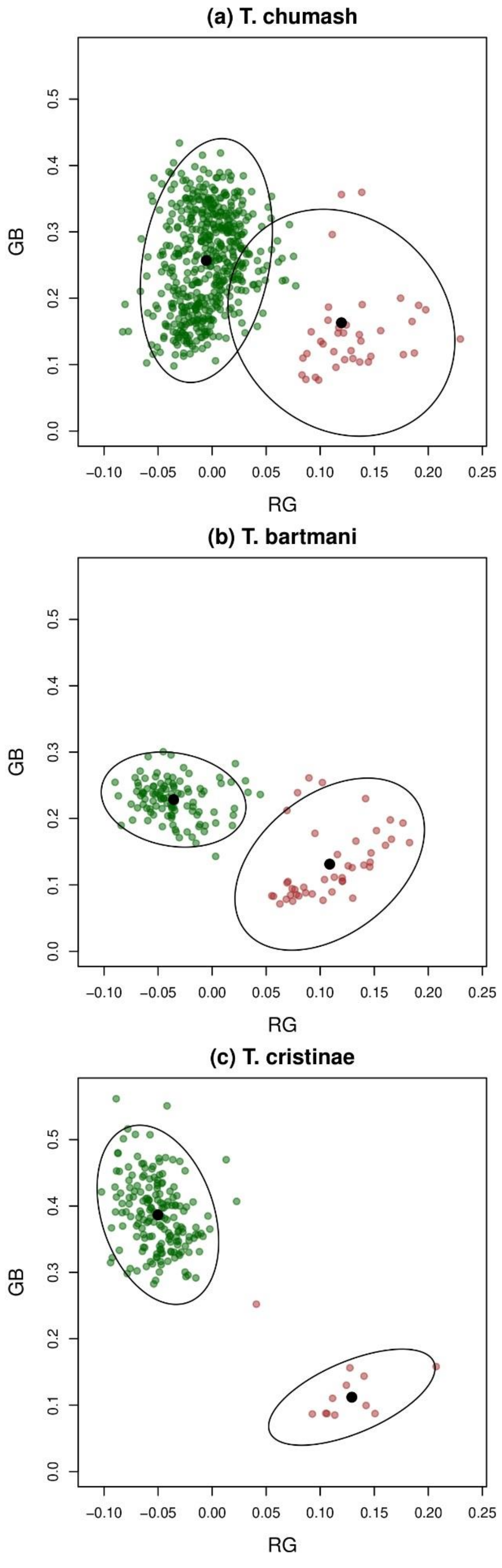
(a) T. chumash
(b) T. bartmani
(c) T. cristinae
GB
RG
0.0
0.1
0.2
0.3
0.4
0.5
−0.10
−0.05
0.00
0.05
0.10
0.15
0.20
0.25

Fig. S5. Evidence of bimodality of *Timema* coloration from mixture models.

Panels shows empirical *Timema* color data, where the green and brown dots are clusters corresponding to green versus melanistic morphs. Elipses are 95% expectations from the best fitting models including two bivariate normal distributions, one corresponding to each morph. Large, black dots denote the means of these distributions.

Table S1. AIC values comparing model fit for one versus a mixture of two bivariate normal distributions for color.

Species	AIC one distribution	AIC two distributions
T. bartmani	-871.7426	-1027.618
T. chumash	-3067.743	-3419.47
T. cristinae	-1165.094	-1383.088

Table S2. Contingency table to test the null hypothesis that relative survival of intermediates in the experiment did not depend on host plant treatment. Nb. int. recapt. = number of intermediate individuals recaptured. Nb. non-int. recapt. = number of non-intermediate individuals recaptured. MM = *Cercocarpus* sp. treatment. AC = *Adenostoma* sp. and *Ceanothus* sp. treatment.

Treatment	Nb. int. recapt.	Nb. non-int. recapt.
MM	13	13
AC	2	14

Table S3. ***Timema*** **populations used for spectral reflectance measurements.**

For *T. cristinae*, 5 specimens of each morph were randomly sampled for reanalysis. Pop. Code = population code. Host-plant abbreviations are as follows. A: *Adenostoma fasciculatum*, MM: *Cercocarpus* sp., Q: *Quercus* sp., WF: *Abies concolor*, WP: *Pinus* sp. Morph = color morph. N-ind = number of *Timema* individuals used for this analysis.

**Species**	**Pop. code**	**Host plant**	**Morph**	**N-ind**
T. bartmani	JL	WF, WP	green	4
T. bartmani	JL	WF, WP	melanistic	5
T. chumash	GR8.06	MM	green	4
T. chumash	GR8.06	MM, Q	melanistic	6
T. cristinae	FH	A	green	5
T. cristinae	FH	A	melanistic	5

Table S4. Host-plant samples used for spectral reflectance measurements.
N-samples = number of plant samples used for this analysis.

**Host plant**	**Plant part**	**N-samples**
Abies concolor	upper leaf	7
Abies concolor	lower leaf	5
Abies concolor	stem	3
Adenostoma fasciculatum	needles	5
Adenostoma fasciculatum	stem	5
Ceanothus spinosus	upper leaf	10
Ceanothus spinosus	lower leaf	10
Ceanothus spinosus	stem	4
Cercocarpus montanus	upper leaf	9
Cercocarpus montanus	lower leaf	9
Cercocarpus montanus	stem	4

Table S5. ***Timema*** **populations used for coloration measurements from digital photographs.** Pop. code = population code. Host-plant abbreviations are as follows. A: *Adenostoma fasciculatum*, MM: *Cercocarpus* sp., Q: *Quercus* sp., WF: *Abies concolor*, WP: *Pinus* sp.. Lat. = latitude. Long. = longitude. N-ind = number of *Timema* individuals used for this analysis.

**Species**	**Pop. code**	**Host plant**	**Lat.**	**Long.**	**N-ind**
T. bartmani	JL	IC, WF, WP	34.16	-119.9	150
T. chumash	GR8.06	MM, Q	34.22	-117.71	541
T. cristinae	FH	A	34.52	-119.8	190

Table S6. Host-plant samples used for coloration measurements from digital photographs. Pop. code = population code. Lat. = latitude. Long. = longitude. N-pictures = number of pictures used. N-samples = number of plant samples measured for this analysis.

Host-plant species	Pop. code	Lat.	Long.	N-pictures	N-samples
Adenostoma fasciculatum	DZR	33.86	-116.83	16	102
Ceanothus spinosus	NH	34.52	-119.8	13	86
Cercocarpus sp.	GR	34.22	-117.71	33	98
Quercus sp.	SM	37.02	-121.73	28	99
Quercus sp.	GR	34.22	-117.71	39	89
Quercus sp.	BC	36.06	-121.57	41	99
Abies concolor	BM	33.84	-116.75	17	101
Pinus sp.	JL	34.17	-119.9	16	87
